# Cases of Peripneumonic Abscess of the Lungs; with Some Remarks

**Published:** 1827-09

**Authors:** W. F. Chambers

**Affiliations:** Physician to St. George's Hospital.


THE LONDON
### Medical and Physical Journal.

No. 343, VOL. LVIII.] SEPTEMBER, 1827. [No. 15, *New Series*.

For many fortunate discoveries in medicine, and for the detection of numerous errors, the world is indebted to the rapid circulation of Monthly Journals; and there never existed any work, to which the Faculty, in Europe and America, were under deeper obligations, than to the Medical and Physical Journal of London, now forming a long, but an invaluable, series.—RUSH.

### ORIGINAL PAPERS,

AND

### CASES OBTAINED FROM PUBLIC INSTITUTIONS AND OTHER AUTHENTIC SOURCES.

### PULMONARY ABSCESS.

*Cases of Peripneumonic Abscess of the Lungs; with some Remarks.*

By W. F. CHAMBERS, M.D. Physician to St. George's Hospital.

WE are indebted to the pathologists of the present age for our knowledge of the fact that suppuration of the lungs, consequent on simple inflammation, is a disease of exceedingly rare occurrence. Dr. DUNCAN, sen. in his Treatise on Consumption, published in 1813, makes apostema of the lungs one of the species of phthisis pulmonalis; and, although he states that it is a less frequent modification of the disease than that which arises from bronchial inflammation or tuberculous concretion, he does not seem to have been fully impressed with its very great comparative rarity; nor, indeed, am I aware that any writer before LAENNEC has placed this matter in a just point of view.

The truth is that what has long been known with reference to another organ, the liver, is almost equally applicable to the lungs. I mean that, as in the liver, it is infinitely more common, in this country at least, to find that inflammatory action has proceeded just so far as to cause the deposit of adhesive or indurating matter in its substance, than that it has been intense enough to induce suppuration; so in the lungs, when inflammation takes place, in a large majority of cases, it stops short of suppuration, producing, for the most part, induration or hepatisation of the affected portion of the viscus. Hence, then, it is easy to account for the comparative rarity of apostematous phthisis. For, as it will be allowed that tuberculation of the lungs is a much more frequent affection than their simple phlegmonous inflammation, and as we have seen also that, even when the latter occurs, it is very seldom of such intensity as to destroy the affected part by suppuration, whilst the former almost invariably leads to the formation of vomica; it follows, of course, that phthisis consequent on peripneumonic suppuration must be a much less frequent disease than that which arises from the maturation of tubercles in the lungs.

I may be allowed to say here, also, that, during the time I have been attached to St. George's Hospital (a term of nearly fifteen years), my own experience justifies this conclusion. Within the above-mentioned period, the number of deaths from pulmonary disease has amounted to above 600. The bodies of all these persons, with a very few exceptions, have been carefully examined after death, and amongst them, according to the best of my recollection, there have been found only three cases of pure phlegmonous abscess in the lungs.* During this period also many individuals, with diseases of the lungs, have left the hospital, after receiving various degrees of relief. Amongst these, three or four instances of pulmonary apostema have perfectly recovered.

We may say, then, that, out of many hundred cases of pulmonary disease, only six or seven have occurred of true phlegmonous abscess; of which it may be remarked, that at least one-half have terminated favourably.

Having premised these observations, I will now relate two cases of the disease in question, taken from the journals of the hospital. The first of these, it will be seen, terminated in recovery. The second died in the house, and afforded us an opportunity of examining the diseased organ; the appearances of which verified the opinion which the previous symptoms had suggested respecting the true nature of the

* In this number, I do not include those appearances of circumscribed abscess discovered by dissection in persons who have died after severe injury of the head, or any other great disturbance of the nervous system, which sometimes occur not only in the lungs, but in the liver, in the spleen, and, in fact, in almost all glandular and cellular parts of the body. These are to be placed in the same class as the collections of albuminous matter in the serous cavities, which are frequently found after death, under the circumstances just mentioned. All of these, in spite of their similarity to the results of inflammation, owe their origin to a process essentially distinct from common phlegmonous action. They are in some way the consequence of the total disorder subsisting in the nervous and secretory systems, and, when they appear in this manner in the lungs, must not be confounded either with the effects of ordinary peripneumony or of tuberculation.

malady, and rendered it probable that we were right also with regard to the complaint of the first-mentioned individual who recovered, inasmuch as the features of the disease were the same in both.

Case I.—Francis Brennin, aged thirty-four, labourer, of St. James's parish, admitted January 5th, 1825, of a muddy, sallow complexion, and much emaciated. He complains of difficulty of breathing, with heavy pain in the upper part of the left side of the thorax, which is less expansible than the right; he has a very troublesome cough, with a copious expectoration of a brownish yellow colour and *most offensive smell*, tinged with dark-coloured blood; *his breath also has a putrid smell*, which can be perceived at some distance. He cannot lie on either side, on account of his cough; and he complains besides of a dull pain in his head. He has no regular hectic, but has occasional shiverings, with flushings, followed by perspiration. Pulse 130, small; skin cool; tongue of a livid colour, with a smooth, clean, moist surface; bowels open; urine high coloured; appetite indifferent.

He says that some months ago he received a blow on his left side, ever since which he has suffered more or less aching in the part, but that within the last month he has been attacked with severe pain there, accompanied by fever, cough, and headache, and subsequently with expectoration. He states that he has been treated with cupping and blisters, which have considerably diminished the severity of the pain.

He was now ordered to be placed on milk-diet, and to take the following medicines:—Haust. Salin. cum Mucilag. Acaciæ ʒj.; Tinct. Digital. m. v.; Vin. Ipecac. m. xv. sextâ quâque horâ.—Extr. Colocynth. comp., Extr. Lactucæ sativæ, āā gr. v. omni nocte.

Under this treatment he daily improved until the 21st. The report on that day is as follows:—Cough diminished; expectoration less in quantity, but still fetid; less pain in the side; pulse natural; skin cool; bowels regular. He still sweats occasionally at night.

Mist. Ferri comp. ℥j.; Mist. Camphoræ ℥ss. ter die.—Rep^r pilulæ.—(Meat daily, with a pint of porter.)

This treatment was continued with little variation until the 14th of March. He had then gained flesh, he coughed very little; his expectoration was of a lighter colour, had scarcely any smell, and was much diminished in quantity. His pulse, tongue, skin, bowels, and urine, were in a natural state; but still the left side of the thorax was not perfectly expanded by inspiration. He was now directed to attend as an out-patient until the autumn, when I discharged him, perfectly restored to health. It was remarkable that the left lung at last entirely regained its expansibility.

I will reserve the few remarks which are suggested by the history of this case until I have related another instance of the same disease which terminated unfavourably, and afforded us an opportunity of examining its seat after death.

John Hayward, aged forty-five, labourer, of St. James's parish, admitted October 4th, 1826, of a sallow complexion, much emaciated, and very weak. He complains of difficulty of breathing, cough, and expectoration of considerable quantities of foul, *highly offensive*, purulent matter, of a dark yellow colour, tinged in part with blood. *His breath is very fetid;* he has pain in his chest on both sides, which is aggravated by any attempt to make a deep inspiration. He lies generally on his back. Pulse 110, small, but somewhat sharp; skin hot; tongue red at the edges, furred in the middle; bowels open; urine high-coloured; appetite indifferent.

He says he was attacked eleven weeks ago with symptoms which he describes as those of peripneumony, but was not bled for them. These subsided in a fortnight, and were followed by the expectoration above described, and the other present symptoms.

He was placed on fever diet, and ordered to lose eight ounces of blood from the arm, and to take the following medicines:—Haust. Salin. cum Liq. Antim. Tartar. m. xvj. sextâ quâque horâ.—Magnes. Sulphat. ℥ ss. cràs.

October 5th.—Blood cupped and buffy. Pain relieved; pulse eighty, soft; skin cooler; tongue clean; cough less frequent; bowels open.

Repetantur medicamenta.

He remained nearly in this state until the 8th, when a bowel-complaint occurred. This was allayed by small doses of Dover's power; but, on the night of the 14th, his strength seemed to fail him altogether. He was ordered now to take from time to time small quantities of wine or brandy, mixed with warm water. He did not, however, rally, but died on the following day.

The body was examined on the 16th, and presented the following appearances of disease:

The lower lobe of the left lung was somewhat indurated towards the posterior part. In the right side, there were a few ounces of opaque serum in the pleura. The apex of this lung was enveloped in thickened pleura, which adhered closely at that part to the contiguous pleura costalis. On cutting into the lung, two cavities, each about the size of a small orange, were brought into view; one in the superior part of the upper lobe, the other in the centre of the middle lobe. The first was lined with a loose sloughy substance, and filled with dark yellow fetid matter; the second was empty, from the pus and sloughy matter having been expectorated through the bronchial tubes, which were seen communicating with it, and was lined with a very thin membrane of a dark colour. The rest of the lung was somewhat compressed by the fluid in the pleura, and particularly at the lower part; whilst, in the neighbourhood of the abscesses, it was slightly indurated, or hepatised; but there was no appearance of tuberculation in either lung.

There were no appearances of disease in the abdomen, except an inconsiderable enlargement of a few of the mesenteric glands.

These two cases are, I think, fair specimens of the disease of which we are speaking, and show that its symptoms are pretty clearly distinguishable from those which characterise ordinary tuberculous consumption.

If, indeed, we can ascertain with accuracy the early symptoms of the disease, we shall have little difficulty in fixing on its true nature. This, however, is not always in our power, either from want of observation and intelligence in the patient himself, (especially when he happens to be of the uneducated class of the community,) or else from the insidiousness of true pulmonary consumption, whose earliest approaches are often overlooked, and its commencement dated from some accidental inflammatory attack in the lungs or pleura, which may be in fact altogether a secondary affection.

In this difficulty with respect to the history of the complaint, the symptoms, as observed in the two cases just described, will be generally decisive of its real character.

In the first place, the appearance and the smell of the expectoration are totally different from those which belong to the sputa of tuberculous consumption.

In apostema of the lungs, the expectoration is of a brownish or greenish yellow colour, and has an intense odour of putrefaction. This colour was correctly compared, in my hearing, by a physician of considerable experience, particularly in diseases of the lungs, to that of "rotten eggs;" an appearance in the expectoration which, he said, he had long been accustomed to consider, when joined with fetor, as holding out a more favourable prospect of recovery to the patient than that of ordinary purulent matter. This appearance is obviously owing to the mixture of pus and blood with the particles of sloughy lung, which form the parietes of such abscesses: to the latter also is, of course, to be attributed the offensive odour of the sputa. It is not to be wondered at, under such circumstances, that the breath of the individual should be of an almost intolerable smell.

It may be remarked here, that, in cases which terminate favourably, the dark colour of the expectoration disappears, and its fetor decreases gradually, as the sloughy parietes of the cavity are, from time to time, ejected by coughing.

It is scarcely necessary to point out at length the difference between this kind of expectoration and the white or yellowish inodorous sputa, occasionally streaked with more or less scarlet blood, which characterise tuberculous phthisis. I have said that the latter are generally inodorous: if they be otherwise at any time, it may be considered an accidental occurrence, arising from the confinement of the matter for an unusually long period in a vomica, and they even then retain their ordinary appearance; and are very unlike, in this respect, to the expectoration I have endeavoured to describe as belonging to peripneumonic abscess.

The next distinctive mark of this disease which I will mention is, if I may so speak, a negative one: it is the absence of regular hectic fever, after the abscess is fairly opened into the air-tubes.

In the first of the cases related above, the shiverings, flushings, and perspirations were slight in themselves, and took place only occasionally; whilst, in the second, there was no hectic sweating at all.

Besides this, we may remark that the clear complexion and bright colour, which so often attend tuberculous affection, is absent in cases of apostema of the lungs, in which the complexion is characterised, for the most part, by a dull muddy sallowness.

I am well aware here, also, that there are instances even of true tuberculous consumption in which the complexion is thick and bloated, or in which hectic fever is not observable. In truth, every practitioner must have now and then seen tuberculous consumption proceeding to a fatal termination, without any elevation of the pulse above its natural standard of frequency, or any evident increase of the temperature of the skin. These, however, are exceptions to the general rule, and, when they present themselves to us in practice, are less fertile sources of perplexity than they may at first sight appear to be; inasmuch as our diagnosis is to be at all times founded, not on single symptoms, but on a full and careful observation of all the circumstances of the disease.

We may say, then, that where the expectoration is such as we have described above,—where the breath has a highly putrid odour,—where the hectic fever is indistinct and irregular,—where the complexion is of a sallow or leaden hue,—and where, besides, the history of the case is that of simple inflammation from the commencement; or even where some of these diagnostic marks are deficient or indistinct, whilst the majority of them are clearly present, we may decide, with as much certainty as the general imperfection of our science will allow of in any case, that the disease is apostema of the lungs, and not tuberculous consumption.

Nor, indeed, is our diagnosis to be here considered a matter of mere speculative curiosity; as it is intimately connected with that which is of paramount interest to the patient and to his friends: I mean the prognosis afforded by the disease. For, if the quantity of matter expectorated in such cases as those described above were presumed to be the produce of tuberculous vomica, it is almost unnecessary to say that the prospects of the individual would be unfavourable indeed: whilst, on the contrary, if we have reason to think that the sputa arise from simple suppuration of a part of the viscus, we may look forward with much more sanguineness to the termination of the malady, as undoubtedly a large proportion of such cases arrive, under proper treatment, at a favourable conclusion.

With regard to the treatment of this disease, it will be seen, by a reference to the cases I have related, that the separation of the sphacelated part of the lung, of which the parietes of the abscess or abscesses are formed, is often accompanied, even in an advanced period of the complaint, with attacks of inflammatory action, requiring venesection and the other expedients applicable to the treatment of inflammation; the necessity for which will be sufficiently pointed out by the state of the pulse and the skin, and the aggravation of pain in the seat of the disease. As soon, however, as this stage of the disease has passed by, the patient will be observed to thrive and fatten under the administration of chalybeates and other tonics, and the use of a more nutritious diet than was previously admissible; means which may be resorted to even when the cough is troublesome and the expectoration still purulent and bloody, with much more boldness than would be justifiable under similar circumstances in cases of tuberculous affection of the lungs.

The mode in which the lungs recover themselves, and become capable of again performing their ordinary functions, without any particular inconvenience or difficulty, after they have suffered from simple suppuration, may also be fairly deduced from the cases which I have described. It will be observed in the first of these cases, that during the earlier part of its progress, and even after the disease had taken a favourable turn, the left lung, in which the abscess occurred, was less expansible than the right, in which it may be presumed there was no disease; so that, when the patient drew in his breath deeply, the right side was much more elevated by the inspiration than the left; but that, as the fetid matter was from time to time ejected, and the diseased lung freed from the suppurated and sloughy parts, it gradually recovered its expansibility, and at length appeared to admit air as freely as the other lung.

Now the manner in which this was effected is shown by the dissection of the case which died. In it one of the cavities still contained sloughy and purulent matter; but the other, the inferior one, had been cleared out, and was found lined with a smooth membrane, very similar to, although of a darker colour than, the ordinary mucous lining of the airtubes, and the cavity had evidently, during the latter part of the patient's life, served the purpose of a large air-cell.

In the same manner we may presume that the *whole* of the excavation in the case of Brennin (who recovered) was emptied of its contents, and rendered capable of admitting air, and thus at length allowing the lung to be expanded as usual during inspiration.*

We must recollect, besides, that as, during the inflammation and suppuration of a portion of the lung, some of the neighbouring pulmonary tissue, although not absolutely involved in the suppurative process, may become indurated by the deposition of coagulable lymph; so if, after a time, the lymph thus deposited is removed by the absorbents, which I believe to be possible when the hepatisation is not extensive, this impediment also to the free dilatation of the chest will be removed.

If it be asked why the former of these cases recovered, whilst the other terminated in death, it may be suggested, in the first place, that the individual who died had been suffering much longer from the disease than the patient who recovered; that the former had not been medically treated at all, whilst the latter had been cupped and blistered before his admission; that, in the case which terminated favourably, the disease was obviously confined to a small portion of the left lung, whilst, in the other instance, both lungs had been more or less inflamed; and that, besides the peripneumony which had partially destroyed the substance of the right lung, the patient had suffered from pleurisy, as evinced by the empyematous effusion in the right cavity of the thorax.

Before I conclude, I will take the opportunity of saying a very few words on the subject of common tubercles of the lungs. In the above remarks, I have often mentioned tuberculous vomica as an ordinary and well-known form of degeneration in the pulmonary tissue. Now the question respecting the true pathology of this variety of tubercle, has given rise to many ingenious attempts towards its elucidation. I trust, however, I shall not be considered presumptuous in saying that no one of the explanations hitherto offered, as far as I am acquainted with them, appears to me to be conclusive on the subject. My own impression, indeed, founded on repeated examinations of these bodies, and a careful consideration of all their varieties and modifications, has long been that the original nuclei of pulmonary tubercles are formed in the

* A curative process, somewhat similar to this, is occasionally instituted in tuberculous excavations of the lungs; a distinct instance of which we have lately seen in the dissecting-room of St. George's Hospital.

mucous glands or follicles of the membrane lining the extreme air-cells and air-tubes; which species of glands I believe, with Bichat, to abound wherever there is mucous membrane, although they are in many places not discoverable until they are demonstrated by disease; these glands or follicles being choked up with their own coagulated secretion, and, where several of them are similarly gorged and enlarged, often coalescing with each other, become, as I conceive, the sources of irritation and subsequent ulceration of the surrounding tissue. At length this mass of coagulated and vitiated mucus, mixed with the pus of the ulcerative process, is thrown up by coughing, and leaves those cavities in the lungs, lined with a thick, whitish, coriaceous membrane, so well known as tuberculous vomicæ.

I am well aware that there are some obvious difficulties in this explanation of the disease in question; but I am prepared to show, and may perhaps take an opportunity of doing so in a future Number of your Journal, that no view hitherto taken of the origin and progress of these concretions is open to fewer tenable objections than this, or accounts more consistently with the pathology of mucous membranes in general (all of which are subject to a similar disease,) for the phenomena which are observed to accompany the maturation, as it is called, of tubercles in the lungs.

---

